# Irradiation Effects of As-Fabricated and Recrystallized 12Cr ODS Steel Under Dual-Ion Beam at 973 K

**DOI:** 10.3390/ma18143246

**Published:** 2025-07-10

**Authors:** Jingjie Shen, Kiyohiro Yabuuchi

**Affiliations:** 1 National Institute for Fusion Science, Toki, Gifu 509-5292, Japan; 2 Department of Fusion Science, The Graduate University for Advanced Studies, SOKENDAI, Toki, Gifu 509-5292, Japan; 3 Institute of Advanced Energy, Kyoto University, Uji, Kyoto 611-0011, Japan

**Keywords:** ODS steel, recrystallization, dual-ion irradiation, helium bubbles, hardening

## Abstract

The microstructure evolution and hardness variations of as-fabricated and recrystallized 12Cr oxide dispersion strengthened (ODS) steel after dual-ion (6.4 MeV Fe^3+^ and energy-degraded 1 MeV He^+^) irradiation at 973 K up to 10.6 displacements per atom (dpa) at peak damage and 8900 appm He are investigated. Results show that the oxide particles slightly shrink in the as-fabricated specimen, while they are stable in the recrystallized specimen. Furthermore, larger helium bubbles are trapped at the grain boundaries in the as-fabricated specimen, and the size of helium bubbles in the grains is almost the same for both as-fabricated and recrystallized specimens, indicating that reduction of grain boundaries would reduce the potential nucleation sites and suppress the helium segregation. Moreover, no obvious hardening occurs in the as-fabricated specimen, whereas the hardness increases a little in the recrystallized specimen. Based on the barrier model, the barrier strength factor of helium bubbles is calculated. The value is 0.077, which is much smaller and suggests that helium bubbles seem not to significantly induce irradiation hardening.

## 1. Introduction

Oxide dispersion strengthened (ODS) steel is considered as candidate structural material for blanket application in fusion reactors [1,2,3], where it will suffer from severe neutron irradiation doses up to 150–200 dpa, and helium concentration of ~10 appm/dpa [4], inducing irradiation hardening and helium embrittlement. Based on different design concepts, the maximum required temperature and radiation conditions are different, and higher temperatures give rise to higher efficiency. Hence, the stability of oxide particles under high-temperature and neutron irradiation conditions is essential to maintain adequate strength and system soundness for application in nuclear reactors. Many studies have been conducted to reveal helium irradiation effects on microstructure and mechanical properties of ODS steels [5,6,7,8,9,10,11,12,13,14,15,16]. Edmondson et al. [5] implanted helium ions into ODS steels at 673 K and analyzed helium bubble distributions on various interfaces, such as coarse precipitates, dislocations, grain boundaries and oxide particles, showing the oxide particles taking the dominant position. Moreover, some investigations [7,8,13] also demonstrated high density oxide particles can provide abundances of interfaces for trapping helium and effectively suppress the coarsening of helium bubbles. Song et al. [11] reported that even though oxide particles can enhance the swelling resistance of ODS steels, swelling percentage increased with increasing irradiation temperature from 573 K to 973 K. Furthermore, refining the grain size from several hundred nanometers to tens of nanometers leads to outstanding helium bubble suppression [14,15].

ODS steel is fabricated by powder metallurgy, including mechanical alloying, consolidation by hot extrusion/isostatic pressing, and the subsequent thermomechanical process. This process produces fine and elongated grains with a high density of nanoscale oxide particles and dislocations, and preferential orientations [17,18], which lead to low ductility and obviously anisotropic mechanical properties [19,20,21,22]. To enhance the ductility and suppress the anisotropy, a recrystallization process is suggested [23]. A novel thermomechanical process [24], namely multi-directional cold rolling followed by annealing, was developed to obtain recrystallization for 12Cr ODS steel. The improvement of ductility was confirmed for the recrystallized 12Cr ODS steel. Although it can be expected that irradiation hardening and swelling will become more evident because dislocation density and grain boundaries dramatically decreased after recrystallization compared with the as-fabricated state, the response of recrystallized 12Cr ODS steel under irradiation is still not clear. Furthermore, the recrystallization process changes the extremely complex microstructures into simple ones, reducing the high density of dislocations to almost free and obtaining coarse grains. This makes it possible to focus on the stability of oxide particles under irradiation excluding the effects of other defects, such as dislocations and grain boundaries. In addition, it offers a clear microstructure to observe irradiation-induced defects, characterize the interactions between oxide particles and irradiation-induced defects in future research, and finally provides fundamental insights for understanding the underlying mechanisms of oxide particle stability under irradiation. In the present study, we investigate the microstructure changes and irradiation hardening of the as-fabricated and recrystallized 12Cr ODS steel under Fe^3+^ and He^+^ ion irradiation at 973 K.

## 2. Material and Methods

**Figure 1:**
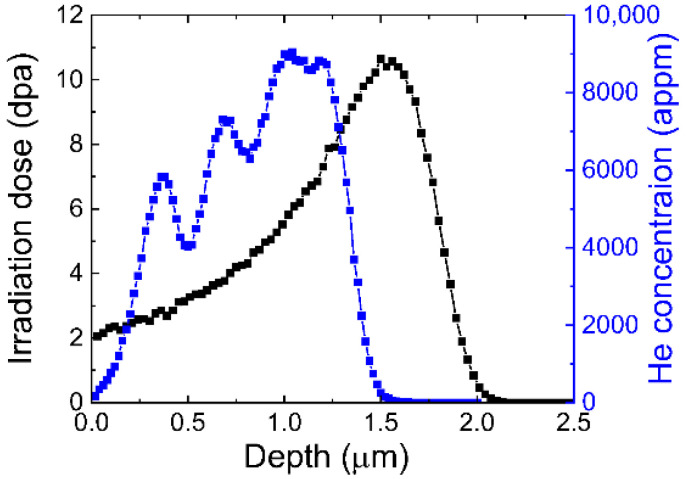
SRIM results show irradiation damage dose and helium concentration as a function of depth.

In this study, 12Cr ODS steel with nominal composition of Fe-12Cr-2W-0.3Ti-0.25Y_2_O_3_ (wt%) was used. It was consolidated by hot extrusion at 1423 K. Then, hot forging was performed at 1423 K followed by annealing at 1373 K for 1 h. Afterward, cold rolling was conducted with 40% thickness reduction and final annealing was carried out at 1323 K for 1 h. Regarding the recrystallization process, a 3 mm thick specimen was cut from the as-fabricated plate. Then, cold rolling was applied on the original normal direction (ND)–transverse direction (TD) section with a 90% thickness reduction. Next, 3 mm diameter discs were punched out from the cold rolled sheet and annealed at 1373 K for 3 h in a vacuum. Before ion irradiation experiments, the as-fabricated and recrystallized specimens were mechanically ground with abrasive paper and electro-polished on one side by Struers Tenupol-5 in an electrolyte of 5 vol% perchloric acid and 95 vol% acetic acid at room temperature. Irradiation experiments were performed at 973 K via Dual-Beam Facility for Energy Science and Technology (DuET) at Kyoto University, using dual-beam 6.4 MeV Fe^3+^ and energy-degraded 1 MeV He^+^ with a flux of 6.37 × 10^15^ and 6.76 × 10^16^ ions m^−2^ s^−1^, respectively. Figure 1 shows the stopping and range of ions in matter (SRIM) calculation results. The irradiation dose is approximately 10.6 displacements per atom (dpa) at the damage peak of 1500 nm, and the helium concentration is approximately 8900 appm at the depth of 1000 nm.

After irradiation experiments, nanoindentation hardness tests were carried out up to a depth of 250 nm from the surface by a DUH-211S hardness tester (SHIMADZU, Kyoto, Japan). The indentation depth was selected from the effective depth where the plastic deformation zone can expand to ~4–5 times the depth of the nano-indenter [25]. The peak helium concentration was located around 1000 nm. It can be noted that Ha et al. [10] used the same facility for the irradiation experiments and found the maximum hardening at 250 nm. The average value was obtained by measuring 80 points for each specimen. The microstructure was characterized by JEM-2800 transmission electron microscope (TEM, JEOL, Tokyo, Japan) at an accelerating voltage of 200 kV. TEM specimens were prepared from the surface of the irradiated specimens by FB-2100 focused ion beam (FIB, HITACHI, Tokyo, Japan) system with Ga ions at 40 kV. To remove the layer damaged by Ga ions during FIB fabrication, flash electropolishing was conducted at approximately 238 K in an electrolyte of 10 vol% perchloric acid and ethanol. Counting the thickness fringes at the two-beam conditions was carried out to evaluate the thickness of the observed area. Approximately four TEM images and 930–1070 particles were measured for each condition.

## 3. Results and Discussion

### 3.1. Microstructure Before Dual-Beam Irradiation

**Figure 2:**
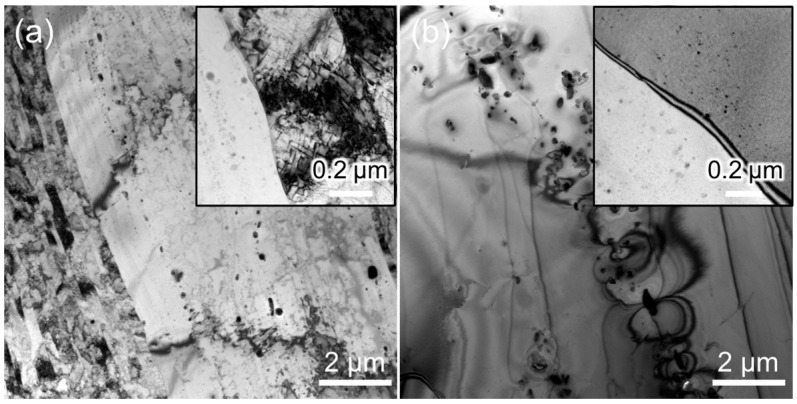
Bright-field TEM images of (**a**) as-fabricated and (**b**) recrystallized 12Cr ODS steel.

**Figure 3:**
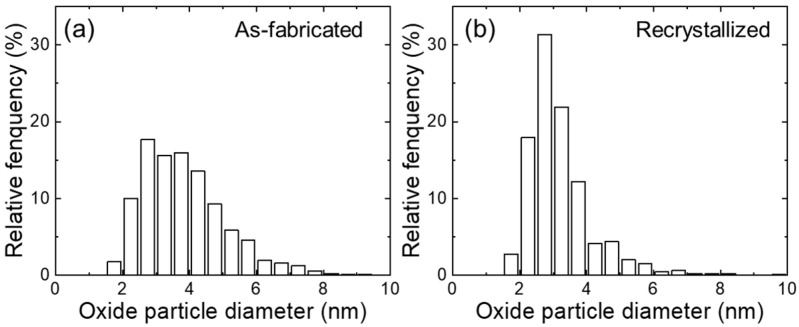
Size distribution of oxide particles in (**a**) as-fabricated and (**b**) recrystallized 12Cr ODS steel.

Figure 2 shows the microstructures of as-fabricated and recrystallized 12Cr ODS steel. For the as-fabricated specimen, non-uniform microstructure is observed, which contains fine and large grains [21]. The fine and elongated grains have a high density of dislocations and a width smaller than 500 nm (see the insert in Figure 2a), whereas the large grains are recrystallized during the fabrication process. Regarding the recrystallized specimen, large recrystallized grains contain much lower density of dislocations and more uniformly distributed nanoscale oxide particles (see the insert in Figure 2b). Additionally, coarse Ti-enriched precipitates are noted in both as-fabricated and recrystallized specimens. Figure 3 shows the size distribution of the oxide particles of as-fabricated and recrystallized specimens. The average diameter and number density of oxide particles in as-fabricated and recrystallized specimens are 3.8 ± 1.2 nm, 3.1 × 10^22^ m^−3^, and 3.2 ± 1.0 nm, 8.1 × 10^22^ m^−3^, respectively. This indicates that the oxide particles become smaller, and more oxide particles are precipitated after the recrystallization process, which are expected to supply more interfaces to absorb irradiation-induced defects.

### 3.2. Microstructure After Dual-Beam Irradiation

**Figure 4:**
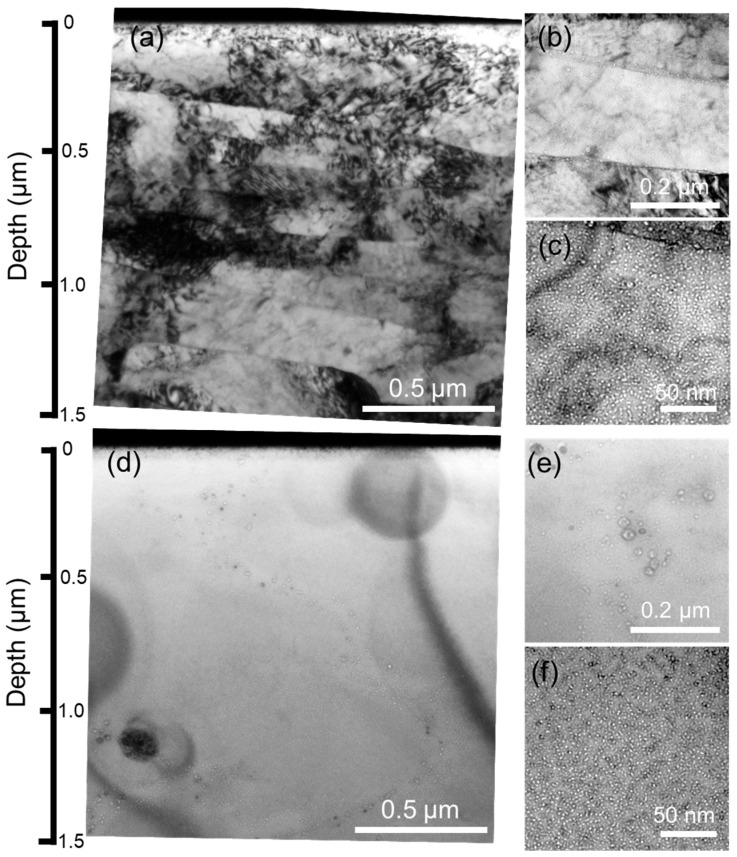
Bright-field TEM images of (**a**–**c**) as-fabricated and (**d**–**f**) recrystallized 12Cr ODS steel after dual-beam irradiation at 973 K. (**a**) Low magnification, (**b**) high magnification showing the bubbles at the grain boundaries and (**c**) in the grains; (**d**) Low magnification, (**e**) high magnification showing the bubbles trapped at the large oxide particles and (**f**) the small oxide particles.

Figure 4 shows the TEM images of the as-fabricated and recrystallized specimens after Fe^3+^ and He^+^ ion irradiation at 973 K. Fine helium bubbles are observed in the irradiated regions. As for the as-fabricated specimen, Figure 4b shows the microstructure near the depth of 1000 nm, where helium bubbles are distributed at the grain boundaries and grain interior with an average diameter of 5.8 ± 1.3 nm and 1.6 ± 0.3 nm, respectively. The number density is ~3.8 × 10^22^ m^−3^. Note that the size of helium bubbles located at the grain boundaries is larger than that at the grain interiors, indicating that helium bubbles preferentially nucleate at the grain boundaries. Helium segregation at the grain boundaries would result in helium embrittlement [26,27,28]. In contrast, even though no grain boundaries are observed in the recrystallized specimen in Figure 4d, helium bubbles are preferentially trapped at the edge of the relatively large oxide particles. Furthermore, helium bubbles located in the grains have an average diameter of 1.7 ± 0.3 nm and a number density of ~3.1 × 10^22^ m^−3^, which are almost the same as the as-fabricated specimen. This suggests that the recrystallization process seems to have no significant effects on helium bubble formation inner grains during Fe^3+^ and He^+^ ion irradiation at 973 K. This can probably be ascribed to more uniformly distributed and finer oxide particle formation after the recrystallization process. In addition, reduction of grain boundaries would decrease the potential nucleation sites and suppress the helium segregation at the grain boundaries.

**Figure 5:**
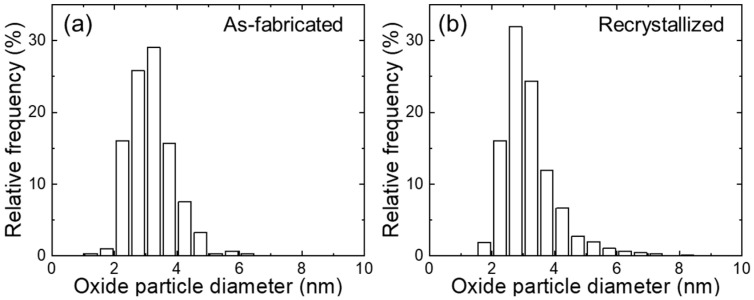
Size distribution of oxide particles in (**a**) as-fabricated and (**b**) recrystallized 12Cr ODS steel after dual-beam irradiation at 973 K.

Figure 5 shows the size distribution of oxide particles in as-fabricated and recrystallized specimens after irradiation. The average diameter is 3.2 ± 0.7 nm and 3.2 ± 0.9 nm for as-fabricated and recrystallized specimens. Compared with the unirradiated specimens, the sizes of oxide particles are slightly smaller for the as-fabricated condition and the same as the recrystallized condition. Allen et al. [29] reported that the size of oxide particles decreased in 9Cr ODS steel at 773–973 K after Ni ion irradiation up to 150 dpa. Song et al. [30] revealed that oxide particles became slightly smaller in Y-Ti-ODS and Y-Al-Zr-ODS after Fe^3+^ and He^+^ irradiation up to 30 dpa at 823 K. Furthermore, in situ TEM study [31] indicated that larger oxide particles (>20 nm) were more evidently affected by ion irradiation than the smaller ones. Slight size reduction of oxide particles is probably caused by ballistic collisions [32,33], and the dissolved atoms more likely diffuse to the dislocations and/or grain boundaries in the as-fabricated specimen than the recrystallized specimen, which contains much lower density of dislocations and grain boundaries.

### 3.3. Irradiation Hardening

**Figure 6:**
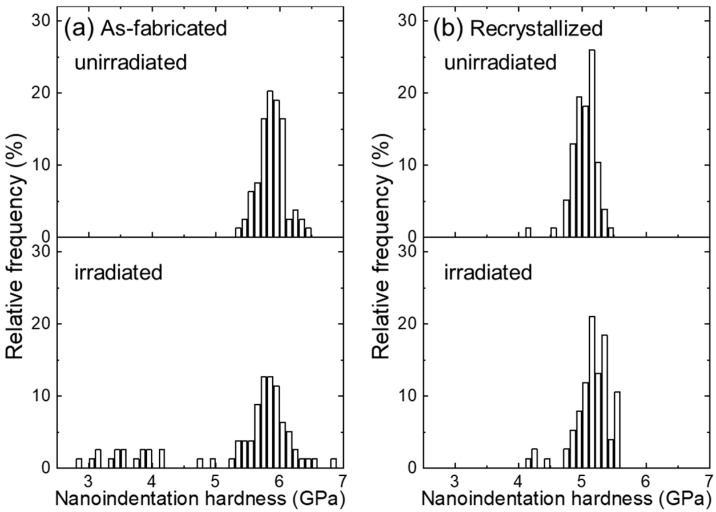
Nanoindentation hardness distribution of (**a**) as-fabricated and (**b**) recrystallized 12Cr ODS steel before and after dual-beam irradiation at 973 K.

**Figure 7:**
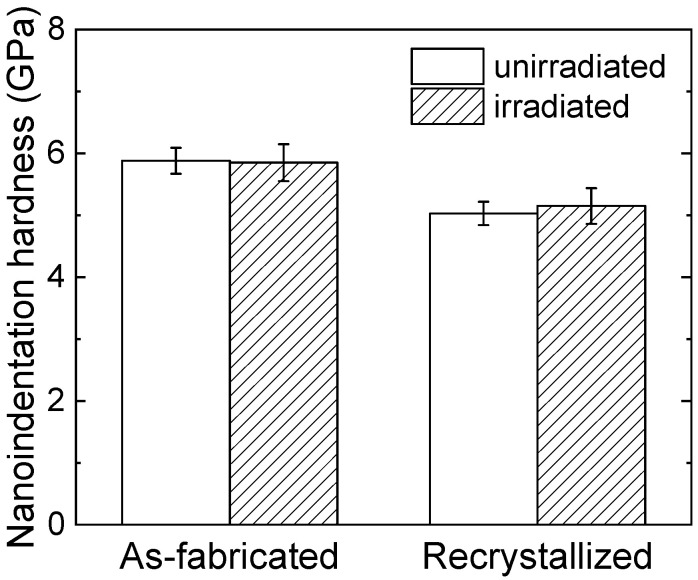
The average nanoindentation hardness of as-fabricated and recrystallized 12Cr ODS steel before and after dual-beam irradiation at 973 K.

Figure 6 shows the nanoindentation hardness distribution in unirradiated and irradiated as-fabricated and recrystallized specimens. As shown in Figure 6a, there are no hardness values lower than 5.0 GPa in the unirradiated as-fabricated specimen, whereas they appear in the irradiated condition. The lower hardness is not due to the irradiation, but non-uniform microstructures. The specimen is fixed on the stage of a nanoindentation hardness tester, and hardness is automatically measured after choosing the test areas. The microstructure cannot be distinguished under the optical microscope of the nanoindentation hardness tester, and the tested area is randomly selected. The lower hardness areas are not tested, whilst they were measured after irradiation experiments. Microstructure is not uniform in as-fabricated specimens. As mentioned in Figure 2, it consists of coarse recrystallized grains and fine grains containing high density dislocations. During thermal annealing for the cold rolled 12Cr ODS steel, recrystallization preferentially occurs at sites containing no/less oxide particles [24]. The lower hardness comes from the recrystallized grains that have no/lower density of oxide particles and already exist in the as-fabricated condition (see Figure 2a). As shown in Figure 6b, the hardness values of recrystallized specimens are around 5.0 GPa. Hence, the values around 3.0~4.0 GPa are measured on the grains containing no/less oxide particles. To examine the hardening effects upon irradiation, these lower hardness values are not considered in comparison with the unirradiated data, and the corresponding average values are shown in Figure 7. In contrast, with respect to the recrystallized specimen, Figure 6b indicates a similar hardness value distribution for unirradiated and irradiated specimens. There are fewer scattered values in the recrystallized condition, suggesting the homogeneous microstructure after recrystallization.

Figure 7 shows the average nanoindentation hardness of unirradiated and irradiated as-fabricated and recrystallized specimens. No obvious hardening is characterized in the as-fabricated specimen, while the hardness slightly increases for the recrystallized specimen after dual-beam irradiation at 973 K. Ha et al. [10] reported that the hardness was increased by 1.079 GPa after Fe^3+^ and He^+^ irradiation up to 90 dpa and 450 appm He at 743 K for recrystallized 15Cr ODS steel, where the oxide particle diameter and number density were 23 nm and 1.9 × 10^21^ m^−3^, respectively. Relatively larger oxide particles with a lower number density may give rise to lower amounts of sinks for the irradiation-induced defects and thus result in more significant irradiation hardening.

The barrier model [34] is usually applied to explain the irradiation hardening by irradiation-induced defects, which is expressed by the following equation:

(1) $$∆\sigma =M\alpha \mu b\sqrt{Nd}$$

where *M* is the Taylor factor (3.06), *α* is the barrier strength, *µ* is the shear modulus (81.6 GPa), *b* is the Burgers vector (0.248 nm), and *N* and *d* are number density and diameter of the defects. The obtained nanoindentation hardness should be converted into yield strength according to the equations [35,36] $∆\sigma =3.06∆H_{V},\;∆H_{V}=94.495∆H_{N}$, where H_V_ is Vickers hardness, and H_N_ is nanoindentation hardness. Assuming the hardening is caused by the helium bubbles after irradiation, the average diameter (1.7 nm) and number density (3.1 × 10^22^ m^−3^) are substituted in the above equations to estimate the barrier strength of helium bubbles. The barrier strength factor *α*_He_ can be determined as 0.077, which is much smaller than other defects, such as precipitates, dislocation loops, and clusters. Therefore, helium bubbles seem not to significantly induce hardening after the dual-beam irradiation at 973 K in this study. Similarly, the helium bubbles are not the predominant contributor to the irradiation hardening after dual-beam irradiation at 743 K [10].

## 4. Conclusions

The microstructure evolution and hardening of as-fabricated and recrystallized 12Cr ODS steel upon dual-ion irradiation at 973 K with 10.6 displacements per atom (dpa) at peak damage and 8900 appm He are studied. The following conclusions can be drawn:

(1) The size of oxide particles slightly decreases in the as-fabricated specimen, while they are stable in the recrystallized specimen, indicating that smaller oxide particles with a higher density after recrystallization supply more sink areas for the irradiation-induced defects.
(2) Similar helium bubbles located at grain interiors are characterized in both as-fabricated (average diameter: 1.6 ± 0.3 nm, number density: ~3.8 × 10^22^ m^−3^) and recrystallized specimens (average diameter: 1.7 ± 0.3 nm, number density: ~3.1 × 10^22^ m^−3^). Larger helium bubbles are observed at the grain boundaries in the as-fabricated specimen, indicating that the grain boundaries are the preferential nucleation sites, and that reduction of grain boundaries would decrease the potential nucleation sites and suppress the helium segregation at the grain boundaries.
(3) Evident hardening is not observed in the as-fabricated specimen, whereas a little hardness increase occurs in the recrystallized specimen. According to the barrier model, the barrier strength factor of helium bubbles is estimated at 0.077, which is much smaller and suggests that helium bubbles do not significantly cause irradiation hardening in this study.

## Data Availability

The original contributions presented in this study are included in the article. Further inquiries can be directed to the corresponding author.
